# Comparative evaluation of the cadaveric, radiographic and computed tomographic anatomy of the heads of green iguana (*Iguana iguana*) *,* common tegu ( *Tupinambis merianae*) and bearded dragon ( *Pogona vitticeps*)

**DOI:** 10.1186/1746-6148-8-53

**Published:** 2012-05-11

**Authors:** Tommaso Banzato, Paolo Selleri, Irene A Veladiano, Andrea Martin, Emanuele Zanetti, Alessandro Zotti

**Affiliations:** 1 Department of Animal Medicine, Production and Health, Clinical Section, Radiology Unit, University of Padua, Viale dell’Università 16, AGRIPOLIS, Legnaro, Padua, 35020, Italy; 2 Clinic for Exotic Animals, Via Sandro Giovannini 53, Rome, 00137, Italy; 3 Department of Comparative Biomedicine and Food Science, Necropsy Room Service, University of Padua, Viale dell’Università 16, AGRIPOLIS, Legnaro, Padua, 35020, Italy

**Keywords:** Green iguana, Tegu, Bearded dragon, Computed tomography, Radiography

## Abstract

**Background:**

Radiology and computed tomography are the most commonly available diagnostic tools for the diagnosis of pathologies affecting the head and skull in veterinary practice. Nevertheless, accurate interpretation of radiographic and CT studies requires a thorough knowledge of the gross and the cross-sectional anatomy. Despite the increasing success of reptiles as pets, only a few reports over their normal imaging features are currently available. The aim of this study is to describe the normal cadaveric, radiographic and computed tomographic features of the heads of the green iguana, tegu and bearded dragon.

**Results:**

6 adult green iguanas, 4 tegus, 3 bearded dragons, and, the adult cadavers of : 4 green iguana, 4 tegu, 4 bearded dragon were included in the study. 2 cadavers were dissected following a stratigraphic approach and 2 cadavers were cross-sectioned for each species. These latter specimens were stored in a freezer (−20°C) until completely frozen. Transversal sections at 5 mm intervals were obtained by means of an electric band-saw. Each section was cleaned and photographed on both sides. Radiographs of the head of each subject were obtained. Pre- and post- contrast computed tomographic studies of the head were performed on all the live animals. CT images were displayed in both bone and soft tissue windows. Individual anatomic structures were first recognised and labelled on the anatomic images and then matched on radiographs and CT images. Radiographic and CT images of the skull provided good detail of the bony structures in all species. In CT contrast medium injection enabled good detail of the soft tissues to be obtained in the iguana whereas only the eye was clearly distinguishable from the remaining soft tissues in both the tegu and the bearded dragon.

**Conclusions:**

The results provide an atlas of the normal anatomical and in vivo radiographic and computed tomographic features of the heads of lizards, and this may be useful in interpreting any imaging modality involving these species.

## Background

Nowadays, reptiles are treated at veterinary practices in a context where both reptile and amphibian medical expertise is constantly improving as more advanced knowledge of these species is scientifically validated [1]. Owners now expect and demand more targeted and expert diagnostic testing for their animals, on the basis of these advances [1]. Reptile medicine has in fact stirred a certain interest in recent years, which is illustrated by the numerous publications validating diagnostic, surgical and anesthesiological techniques in reptile patients [2-14].

Radiographic evaluation of the skull and vertebral column is the most economic and readily available imaging modality for the exotic animal clinician [1]. Furthermore, routine access to CT is becoming increasingly common and many specialty practices have a CT scanner on site [1]. Nevertheless, accurate interpretation of radiographic and CT studies requires a thorough knowledge of the gross and the cross-sectional anatomy [15,16].

To the best of our knowledge, no comprehensive description of the radiographic and computed tomographic features of lizard heads is currently available. Therefore, the aim of this study is to describe and compare the normal radiographic and CT features of the head of some of the most popular pet lizards. Green iguanas and bearded dragons are common reptile pets [17,18]; tegus have recently become popular among collectors and breeders, although no official data exist on this topic.

This study has resulted in a series of tables matching the normal anatomic, radiographic and computed tomographic features of these species.

To the best of the authors’ knowledge, an updated, univocal anatomical reference for the species considered in this study is not available; nevertheless, the anatomy of closely related species has been already thoroughly described and several references describing the anatomy of the head of lizards are currently reported [19-28].

## Methods

### Animals

Three species belonging to three different families of lizards were the object of this study: the green iguana (*Iguana iguana*, infraorder *Iguania*, family *Iguanidae*), the common tegu ( *Tupinambis merianae*, infraorder *Scincomopha*, family *Teiidae*) and the bearded dragon ( *Pogona vitticeps*, infraorder *Iguania*, family *Agamidae*).

The heads from adult cadavers of 4 green iguanas (3 females and 1 male, mean length 85.1 ±18.1 cm, mean weight 2824 ± 856 g), 4 tegus (2 females and 2 males, mean length 42.5 ± 15.1 cm, mean weight 1587 ± 785 g) and 4 bearded dragons (1 female and 3 males, mean length 18.1 ± 7.1 cm, mean weight 385 ± 49 g) were dissected for this study. The animals were referred to the Radiology Unit of the Department of Animal Medicine, Production and Health at the University of Padua (Italy) for specialty examination and were euthanized because of advanced clinical conditions. A complete post-mortem gross examination was performed on each lizard and revealed pneumonia in 4 cases (2 iguanas and 2 bearded dragons), egg retention in 3 cases (1 iguana and 2 tegus), diffuse abscesses in 2 cases (1 tegu and 1 iguana); no lesions were evident in 3 cases (1 tegu, 2 bearded dragons).

Additionally, 13 live adult animals – 6 iguanas (4 males and 2 females, mean length 90.8 ± 20.1 cm, mean weight 3298 ± 922 g), 4 tegus (1 male and 3 females, mean length 55.2 ± 22.1 cm, mean weight 2156 ± 655 g) and 3 bearded dragons (3 females, mean length 22.1 ± 5.4 cm, mean weight 455 ± 75 g) – referred to the above facilities for specialty examination were included in the study. The pathologies affecting the animals did not involve the head in any of the cases and no pathological findings were evident at clinical examination of the head. For this reason, the imaging procedures were extended to the head of the animal upon prior consent from the owner ^a^.

### Imaging procedures

Complete radiographic studies of the head, including LL (left lateral), right lateral, VD (ventrodorsal) and dorsoventral projections, were performed on all specimens and live animals. Radiographs were obtained by means of a computed radiography device (Kodak Point of Care CR-360 System- Carestream Health, Inc – Rochester, USA). All radiographs were displayed with a bone-edge slight enhancement filter.

CT examination was performed on all the live subjects. These animals were sedated with sevofluorane (Sevofluorane 100%, Baxter Spa, Rome, Italy) administered through a face mask. All CT studies were performed in a cranio-caudal direction with the animals lying on ventral recumbency. Pre- and post- contrast CT images were obtained in transverse planes by means of a third-generation CT scanner (Tomoscan® LX, Philips Medical Systems, Amsterdam, Holland). The CT parameters were: axial acquisition mode, rotation time of 2.9 s, voltage of 120 kV, amperage of 125 mA, and slice thickness of 1.5 mm. Contrast medium (Optiray® 300 mg/ml, Covidien Spa, Italy) was injected directly into the caudal vein at the dose of 2.2 ml/kg bw. The images were then displayed in a bone tissue window (window length: 500; window width: 2,000) and a soft tissue window (window length: 40; window width: 400). Only post-contrast images have been reported.

### Anatomical dissections

Stratigraphic gross anatomical dissection of the cadaver heads was performed in 2 green iguanas, 2 tegus and 2 bearded dragons. The dissections were performed within 24 h of death in each patient to minimise post-mortem changes.

Two green iguana, 2 Tegu, and 2 bearded dragon cadaver heads were designated for cross-sectional studies. Immediately after death these latter specimens were placed on a plastic support in ventral recumbency with the legs adherent to the body and successively stored into a freezer (−20°C) and kept there until completely frozen. Cross-sectional anatomic dissection was performed by means of an electric band-saw. Contiguous 5 mm sagittal slices were obtained starting at the snout and reaching the cranial portion of the lungs. The slices were cleaned with water, numbered and photographed on the cranial and caudal surfaces.

The individual anatomic structures were first identified in the anatomically dissected and cross-sectioned heads, on the basis of anatomic references, and then matched with the corresponding structures in the radiographs and CT scans.

## Results

Most of the clinically relevant structures of the head were recognised both in cross-sectional and anatomic dissections (Figures 1, 2, 3, 4, 5, 6, 7, 8 and 9).

**Figure 1:**
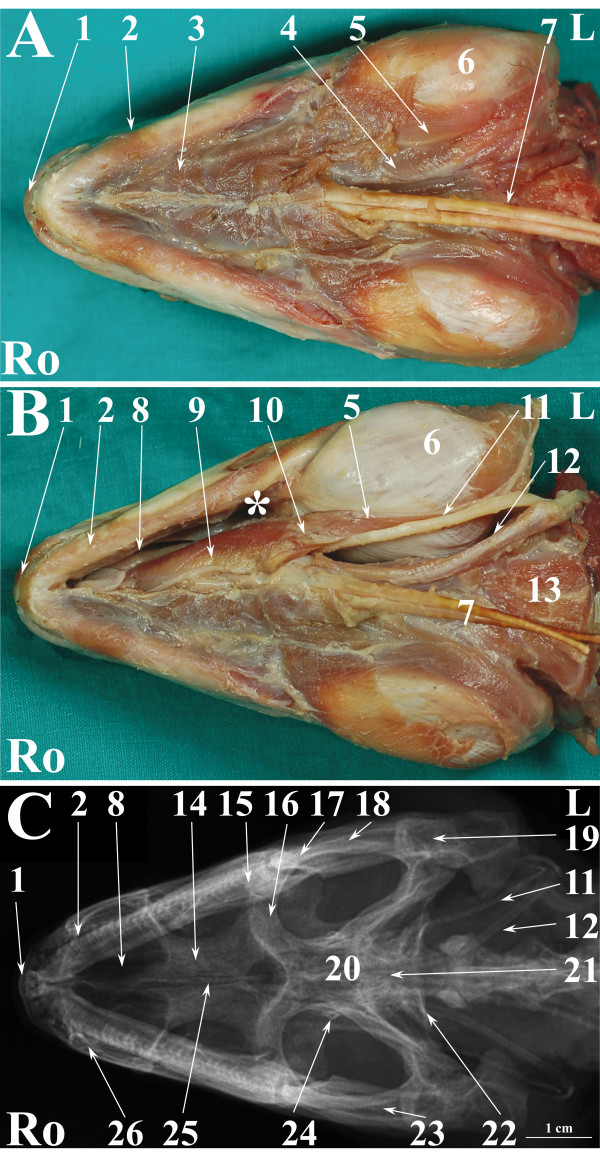
**Normal radiographic anatomy of the head of the green iguana in VD projection. A.** Ventral view in a superficial plane of stratigraphic dissection (only skin was removed) of the head of an iguana. **B**. Ventral view of stratigraphic dissection after removal of Musculus constrictor colli and Musculus intermandibularis (lower part of the image) and a deeper plane of dissection (upper part of the image) of the head of an iguana (deeper plane of dissection is labelled with * on the image). **C**. VD radiographic projection of the head of an iguana. Ro is rostral, L is left.1. Premaxillary bone; 2. Dentary bone; 3. Musculus intermandibularis; 4. Musculus geniohyoideus; 5. Musculus hyoglossus; 6. Musculus pterygoideus typicus; 7. Ceratobranchial 1 process of the hyobranchial skeleton; 8. Vomer bone; 9. Musculus genioglossus; 10. Tendinous band; 11. Ceratohyal process of the hyobranchial skeleton; 12. Ceratobranchial 2 process of the hyobranchial skeleton; 13. Musculus omohyoideus; 14. Palatine bone; 15. Ectopterygoid bone; 16. Pterygoid bone; 17. Jugal bone; 18. Postorbital bone; 19. Articular bone + quadrate bone; 20. Parietal bone; 21. Basioccipital bone; 22. Paraoccipital process of otooccipital bone; 23. Adductor fossa; 24. Epipterygoid bone; 25; Interpterygoid vacuiti; 26. Maxillary bone.

**Figure 2:**
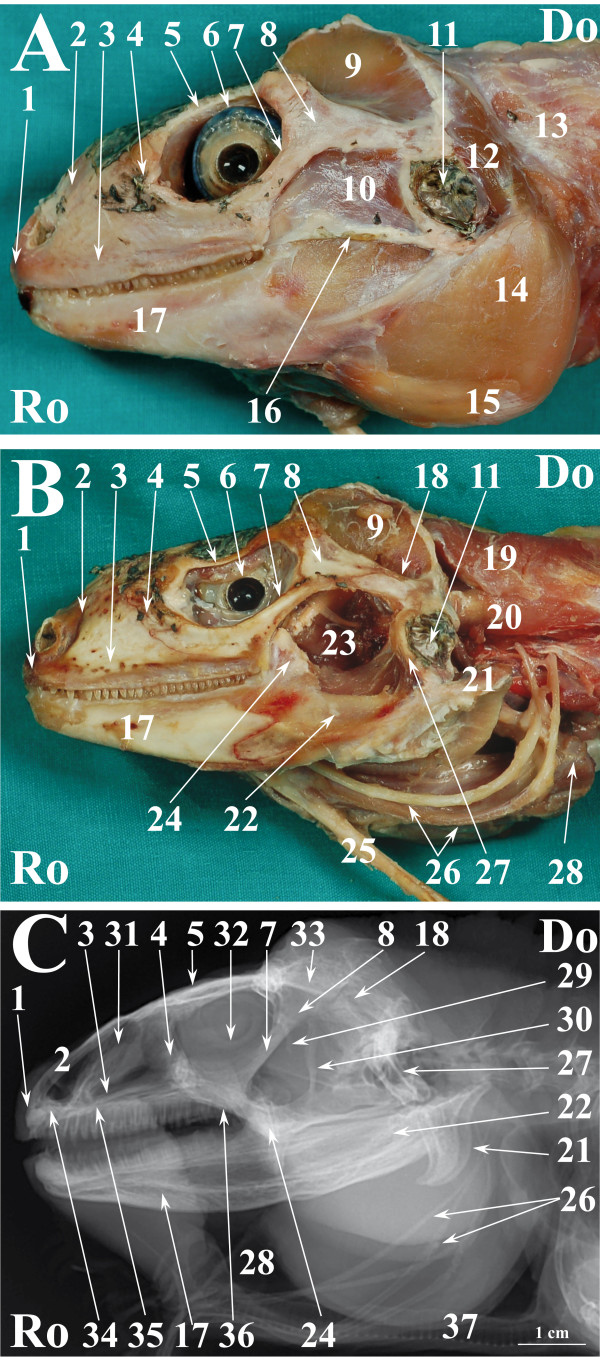
**Normal radiographic anatomy of the head of the green iguana in LL projection.A.** LL photographic image in a superficial plane of the stratigraphic dissection of the head of an iguana (only skin was removed). **B**. LL photographic image in a deep plane of the stratigraphic dissection of the head of an iguana. **C**. LL radiographic projection of the head of an iguana. Ro is rostral, Do is dorsal. 1. Premaxillary bone; 2. Nasal bone; 3. Maxillary bone; 4. Prefrontal bone; 5.Frontal bone; 6. Eye; 7. Jugal bone; 8. Postorbital bone; 9. Musculus adductor mandibulae externus medialis; 10. Musculus adductor mandibulae externus superficialis; 11. Ear; 12. Musculus depressor mandibulae; 13. Musculus episternocleidomastois; 14. Musculus pterygoideus typicus; 15. Musculus intermandibularis posterior; 16. Quadrato-maxillary ligament; 17. Dentary bone; 18. Squamosal bone 19. Musculus trapezius + Musculus clavicle dorsalis; 20. Musculus episternocleidomastoid; 21. Articular bone; 22. Angular bone + Surangular bone; 23. Adductor chamber; 24; Coronoid bone; 25. Ceratobranchial process of hyobranchial skeleton; 26. Hyobranchial skeleton; 27. Quadrate bone; 28. Oesophagus; 29. Pila metoptica; 30. Epipterygoid bone; 31. Nasal glands; 32. Scleral ossicles; 33. Parietal bone; 34. Vomer bone; 35; Palatine bone; 36; Pterygoid bone; 37. Trachea.

**Figure 3:**
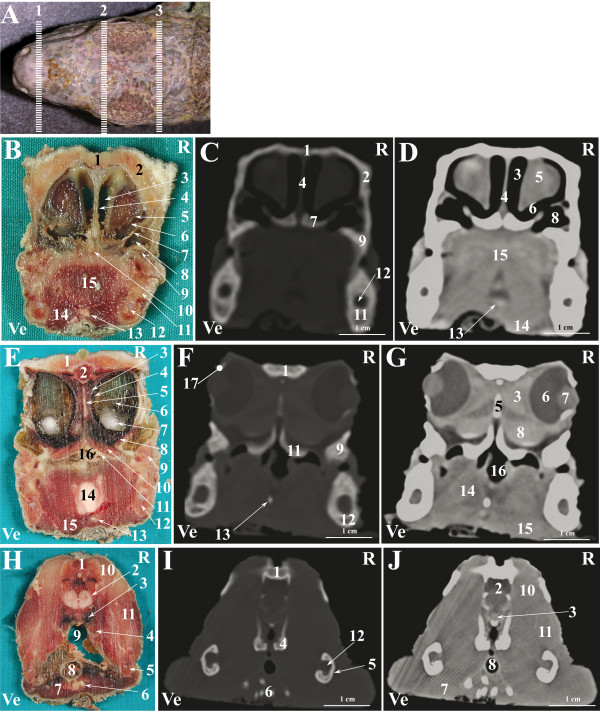
**Normal computed tomographic anatomy of the head of the green iguana.A. **Photograph of the head of an iguana; lines (1–3) indicate the approximate levels of matched cross-sections and CT images. **B**- **J**: Matched images of cross-sectional gross anatomy ( **B**- **E**- **H**), CT image displayed in bone window (WW: +2000; WL: +500) ( **C**- **F**- **I**) and CT image displayed in soft tissue window (WW: +400; WL: +40)( **D**- **G**- **J**) corresponding to: line 1 ( **B**- **C**- **D**), line 2 ( **E**- **F**- **G**) and line 3 ( **H**- **I**- **J**) as depicted in Figure 3A. R is right and Ve is ventral. **B**- **C**- **D**: 1. Nasal bone; 2. Prefrontal bone; 3. Stammteil; 4. Nasal septum; 5. Nasal glands; 6. Subconchal recess; 7.Vomer bone; 8. Choanal tube; 9. Maxillary bone; 10. Cavum oris; 11. Dentary bone; 12. Meckelian fossa; 13. Entoglossal process of the basihyal bone; 14. Musculus geniohyoideus + Musculus hyoglossus + Musculus intermandibularis; 15. Tongue. **E**- **F**- **G**: 1. Frontal bone; 2. Brain; 3. Harderian gland; 4. Choroid plexus; 5. Interorbital septum; 6. Vitreous humor; 7. Lens; 8. Sinus orbitalis; 9. Maxillary bone; 10. Palatine glands; 11. Palatine bone; 12. Dentary bone; 13: Entoglossal process of the basihyal bone; 14. Tongue; 15. Musculus geniohyoideus + Musculus hyoglossus + Musculus intermandibularis; 16. Cavum oris; 17. Scleral ossicles. **H**- **I**- **J**: 1. Parietal bone; 2. Brain; 3. Vena cerebralis media; 4. Sphenoid bone; 5. Angular bone + Surangular bone; 6. Hyobranchial skeleton; 7. Musculus intermandibularis + Musculus genioyhoideus + Musculus hyoglossus + Musculus pterygoideus + Musculus omohyoideus + Musculus stermohyoideus + Musculus ceratohyoideus; 8. Trachea; 9. Oesophagus; 10. Musculus adductor mandibulae externus medialis; 11. Musculus adductor mandibulae externus superficialis; 12. Adductor fossa.

**Figure 4:**
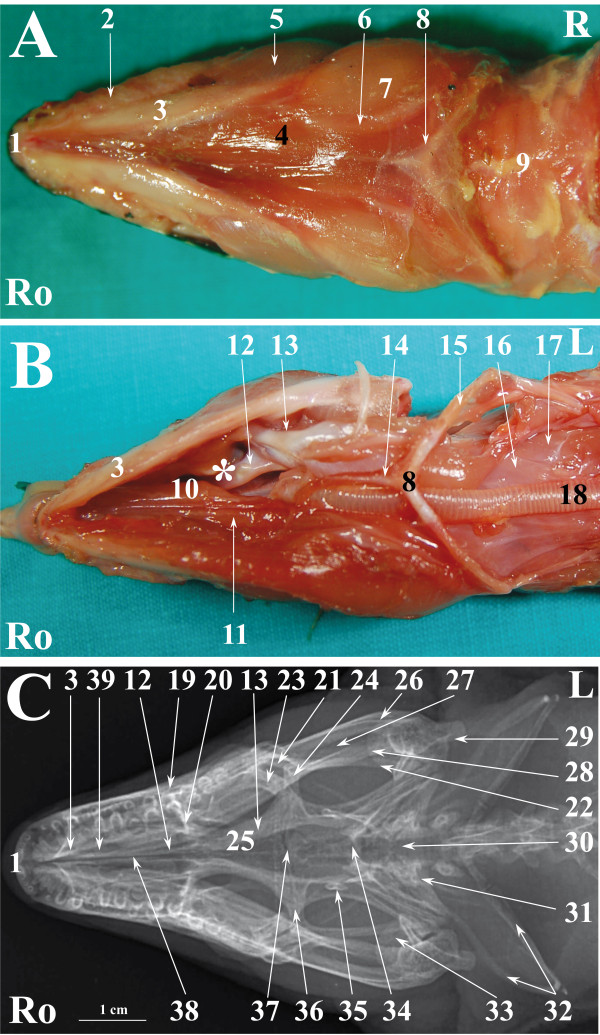
**Normal radiographic anatomy of the head of the common tegu in VD projection.A.** Ventral view in a superficial plane of stratigraphic dissection (only skin was removed) of the head of a tegu. **B**. Ventral view of stratigraphic dissection after removal of Musculus constritor colli and Musculus intermandibularis (lower part of the image) and a deeper plane of dissection (upper part of the image) of the head of a tegu (deeper plane of dissection is labelled with * on the image). **C**. VD radiographic projection of the head of a tegu. Ro is rostral, L is left.1. Premaxillary bone; 2. Infralabial glands; 3. Dentary bone; 4. Musculus intermandibularis; 5. Musculus adductor mandibulae externus superficialis; 6. Musculus hyoglossus; 7. Musculus pterygoideus; 8. Basihyal bone; 9. Musculus constrictor colli; 10. Tongue; 11. Oesophagus; 12. Palatine bone; 13. Pterygoid bone; 14. Entoglossal process of hyobranchial skeleton; 15. Ceratobranchial process of hyobranchial skeleton; 16. Musculus omohyoideus; 17. Musculus sternohyoideus; 18. Trachea; 19. Maxillary bone; 20. Prefrontal bone; 21. Jugal bone; 22. Squamosal bone; 23. Ectopterygoid bone; 24. Postorbital bone; 25. Frontal bone; 26. Surangular bone; 27. Adductor fossa; 28. Angular bone; 29. Articular bone; 30. Basioccipital + Supraoccipital bones; 31. Paraoccipital process of otooccipital bone; 32. Hyobranchial skeleton; 33. Quadrate bone; 34. Parietal bone; 35. Epipterygoid bone; 36. Postfrontal bone; 37. Parieto-frontal suture; 38. Interpterygoid vacuity; 39. Vomer bone.

**Figure 5:**
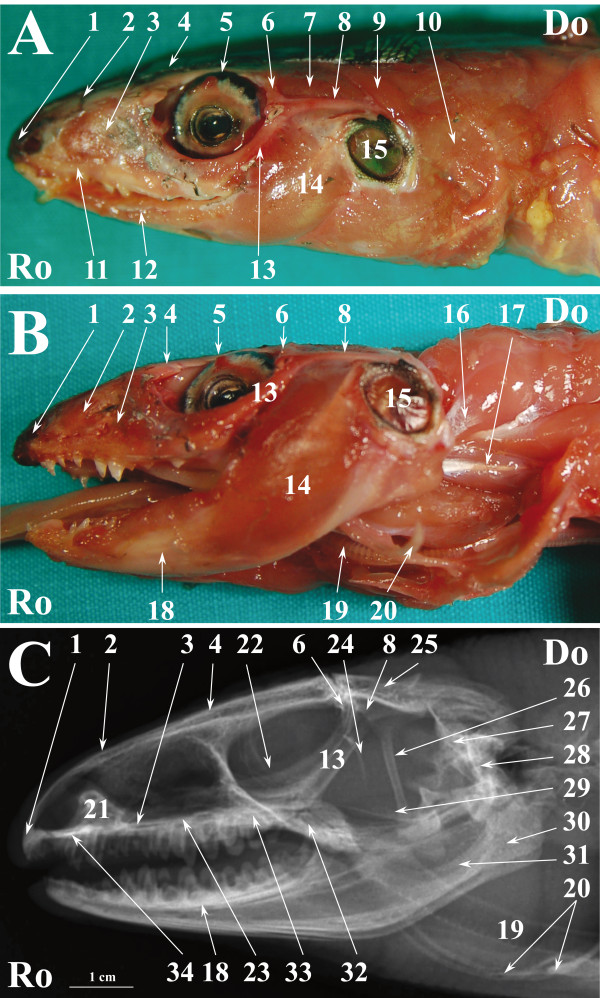
**Normal radiographic anatomy of the head of the common tegu in LL projection.A.** LL photographic image in a superficial plane of the stratigraphic dissection of the head of a tegu (only skin was removed). **B**. LL photographic image in a deep plane of the stratigraphic dissection of the head of a tegu **C**. LL radiographic projection of the head of a tegu. Ro is rostral, Do is dorsal. 1. Premaxillary bone; 2. Nasal bone; 3. Maxillary bone; 4. Frontal bone; 5. Eye; 6. Postorbital bone; 7. Musculus adductor mandibulae externus medialis; 8. Squamosal bone; 9. Musculus depressor mandibulae; 10. Musculus constrictor colli; 11. Supralabial glands; 12. Infralabial glands; 13. Jugal bone; 14. Musculus adductor mandibulae externus superficialis; 15. Ear; 16. Musculus obliquus capitis magnus; 17. Musculus spinalis; 18. Dentary bone; 19. Trachea; 20. Hyobranchial skeleton; 21. Septomaxilla; 22. Scleral ossicles; 23. Palatine bone; 24. Pila metoptica; 25. Parietal bone; 26. Epipterygoid bone; 27. Occipital bone; 28. Quadrate bone; 29. Rostrum parasphenoid; 30. Articular bone; 31. Angular + Surangular bone; 32. Pterygoid bone; 33. Coronoid bone; 34. Vomer bone.

**Figure 6:**
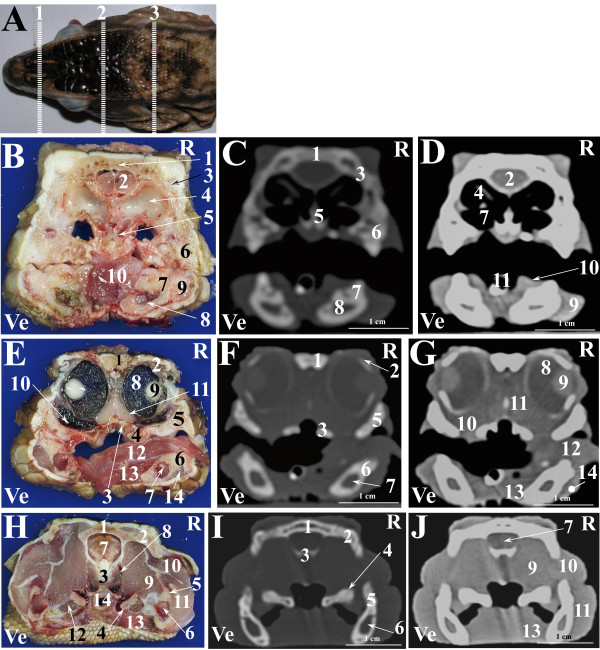
**Normal computed tomographic anatomy of the head of the common tegu.A.** Photograph of the head of a tegu; lines (1–3) indicate the approximate levels of matched cross-sections and CT images. **B**- **J**: Matched images of cross-sectional gross anatomy ( **B**- **E**- **H**), CT image displayed in bone window (WW: +2000; WL: +500)( **C**- **F**- **I**) and CT image displayed in soft tissue window (WW: +400; WL: +40) ( **D**- **G**- **J**) corresponding to: line 1 ( **B**- **C**- **D**), line 2 ( **E**- **F**- **G**) and line 3 ( **H**- **I**- **J**) as depicted in Figure 6A. R is right and Ve is ventral. **B-C-D:** 1. Nasal bone; 2. Nasal glands 3. Prefrontal bone; 4. Nasal conchae; 5. Septomaxilla; 6. Maxillary bone; 7. Dentary bone; 8. Meckelian canal 9. Infralabial glands; 10. Tongue; 11. Trachea. **E**- **F**- **G**: 1. Frontal bone; 2. Scleral ossicles; 3. Palatine bone; 4. Palatine gland; 5. Maxillary bone; 6. Dentary bone; 7. Meckelian canal; 8. Eye; 9. Lens; 10. Sinus orbitalis; 11. Harderian gland; 12. Tongue; 13. Musculus intermandibularis + Musculus geniohyoideus + Musculus genioglossus + Musculus hyoglossus; 14. Infralabial glands. **H**- **I**- **J**: 1. Parietal bone; 2. Squamosal bone; 3. Sphenoid bone; 4. Pterygoid bone; 5. Angular bone + Surangular bone; 6. Adductor fossa; 7. Brain; 8. Musculus longus colli; 9. Musculus adductor mandibulae externus profundus; 10. Musculus abductor mandibularis externus medialis; 11. Musculus intermandibularis posterior; 12. Musculus pterygoideus typicus + Musculus pterygoideus atypicus; 13. Musculus intermandibularis + Musculus geniohyoideus + Musculus pterygoideus typicus + Musculus hyoglossus; 14. Trachea.

**Figure 7:**
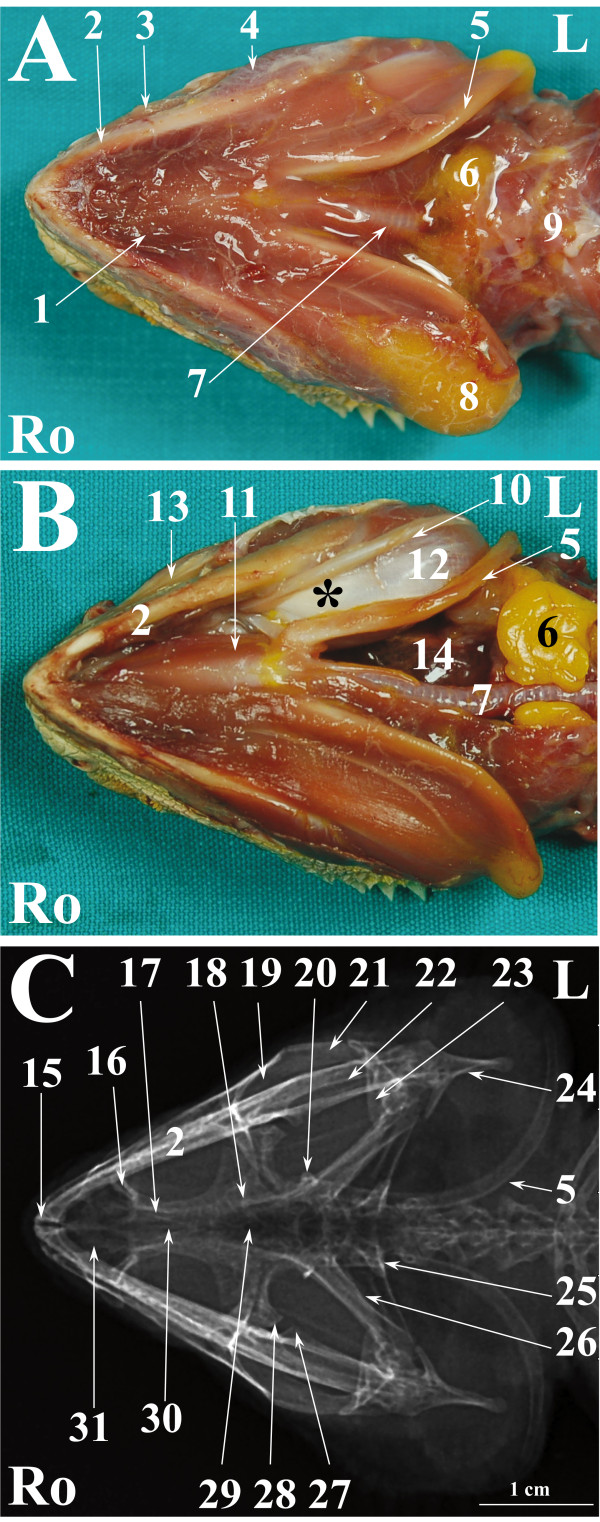
**Normal radiographic anatomy of the head of the bearded dragon in VD projection.A.** Ventral view in a superficial plane of stratigraphic dissection (only skin was removed) of the head of a bearded dragon. **B**. Ventral view of stratigraphic dissection after removal of Musculus constrictor colli and Musculus intermandibularis (lower part of the image) and a deeper plane of dissection (upper part of the image) of the head of a bearded dragon (deeper plane of dissection is labelled with * on the image). **C**. VD radiographic projection of the head of a bearded dragon. Ro is rostral, L is left.1. Musculus intermandibularis; 2. Dentary bone; 3. Supralabial gland; 4. Musculus adductor mandibulae externus supeficialis; 5. Ceratobranchial 1 process of hyobranchial skeleton; 6. Fat body; 7. Trachea; 8. Fat body; 9. Musculus constrictor colli; 10. Ceratohyal process of hyobranchial skeleton; 11. Basihyal bone; 12. Musculus pterygoideus typicus; 13. Maxillary bone; 14. Oesophagus; 15. Premaxillary bone; 16. Prefrontal bone; 17. Palatine bone; 18. Frontal bone; 19. Jugal bone; 20. Epipterygoid bone; 21; Squamosal bone; 22. Angular bone + Surangular bone; 23. Quadrate bone; 24. Articular bone; 25. Paraoccipital process of otooccipital bone; 26. Pterygoid bone; 27. Coronoid bone; 28. Ectopterygoid bone; 30. Interpterygoid vacuiti; 31. Vomer bone.

**Figure 8:**
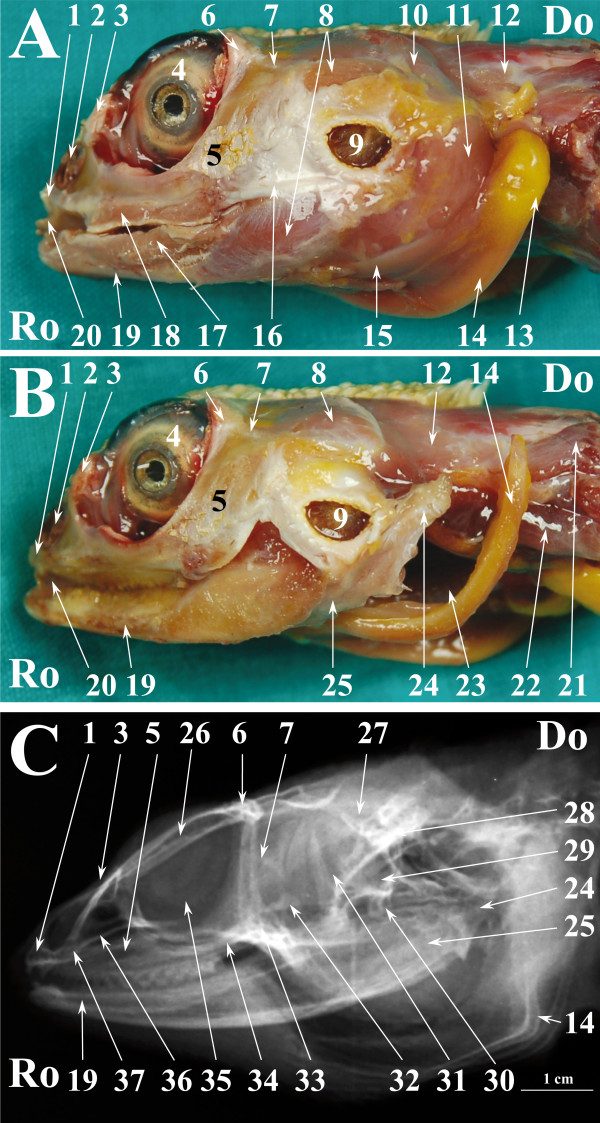
**Normal radiographic anatomy of the head of the bearded dragon in LL projection.A.** LL photographic image in a superficial plane of the stratigraphic dissection of the head of a bearded dragon (only skin was removed). **B**. LL photographic image in a deep plane of the stratigraphic dissection of the head of a bearded dragon. **C**. LL radiographic projection of the head of a bearded dragon. Ro is rostral, Do is dorsal. 1. Premaxillary bone; 2. Nostril; 3. Prefrontal bone; 4. Eye; 5. Maxillary bone; 6. Postorbital bone; 7. Jugal bone; 8. Musculus adductor mandibulae externus superficialis; 9. Ear; 10. Musculus adductor mandibulae externus medialis; 11. Musculus pterygoideus typicus; 12. Musculus depressor mandibulae; 13. Fat body; 14. Ceratobranchial process of hyobranchial skeleton; 15. Musculus sphincter colli; 16. Quadrato maxillary ligament; 17. Infralabial glands; 18. Supralabial gland; 19. Dentary bone; 20. Tongue; 21. Musculus trapezius + Musculus clavicle dorsalis; 22. Musculus episternocleidomastoid; 23. Trachea; 24. Articular bone; 25. Angular bone + Surangular bone; 26. Frontal bone; 27. Parietal bone; 28. Squamosal bone; 29. Paraoccipital process of otooccipital bone; 30. Quadrate bone; 31. Epipterygoid bone; 32. Coronoid bone; 33. Ectopterygoid bone; 34. Pterygoid bone; 35. Scleral ossicles; 36. Palatine bone; 37. Vomer bone.

**Figure 9:**
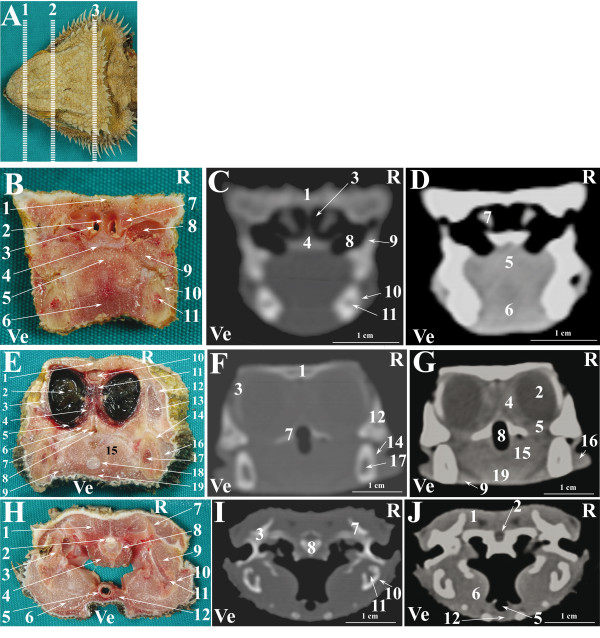
**Normal computed tomographic anatomy of the head of the bearded dragon.A**. Photograph of the head of a bearded dragon; lines (1–3) indicate the approximate levels of matched cross-sections and CT images. **B**- **J**: Matched images of cross-sectional gross anatomy ( **B**- **E**- **H**), CT image displayed in bone window (WW: +2000; WL: +500)(C-F-I) and CT image displayed in soft tissue window (WW: +400; WL: +40) ( **D**- **G**- **J**) corresponding to: line 1 ( **B**- **C**- **D**), line 2 ( **E**- **F**- **G**) and line 3 ( **H**- **I**- **J**) as depicted in Figure 9A. R is right and Ve is ventral. **B**- **C**- **D**: 1. Nasal bone; 2. Nasal septum; 3. Choanae; 4. Vomer bone; 5. Tongue; 6. Musculus intermandibularis + Musculus geniohyoideus + Musculus genioglossus; 7. Nasal glands; 8. Choanal tube; 9. Maxillary bone; 10. Dentary bone; 11. Meckelian canal. **E**- **F**- **G**: 1. Frontal bone; 2. Eye; 3. Scleral ossicles; 4. Harderian gland; 5. Sinus orbitalis; 6. Palatine gland; 7. Pterygoid bone; 8. Cavum oris; 9. Sublingual gland; 10. Brain; 11. Choroid plexus; 12; Postorbital bone + Jugal bone; 13. Musculus adductor mandibulae externus superficialis; 14. Dentary bone; 15. Tongue; 16. Infralabial glands; 17. Meckelian canal; 18. Entoglossal process of the hyobranchial skeleton; 19. Musculus intermandibularis + Musculus geniohyoideus + Musculus hyoglossus + Musculus pterygoideus typicus. **H**- **I**- **J**: 1. Musculus adductor mandibulae externus medialis; 2. Brain; 3. Quadrate bone; 4. Musculus longus colli; 5. Trachea; 6. Musculus pterygoideus typicus; 7. Postparietal process of otooccipital bone; 8. Orbitosphenoid bone; 9. Musculus adductor mandibularis externus superficialis; 10. Angular bone + Surangular bone; 11. Adductor fossa; 12. Musculus intermandibularis + Musculus geniohyoideus + Musculus ceratohyoideus + Musculus hyoglossus.

Figures 1, 4 and 7 show matched images of the ventral view of the stratigraphically dissected heads and VD radiographic projections of the corresponding species. Figures 2, 5 and 8 show matched images of the LL views of the stratigraphically dissected heads and LL radiographic projections of the corresponding species.

Right lateral and dorsoventral radiographic projections were not shown because the radiographic images were identical to the LL and VD projections, respectively.

Figures 1A, 4A and 7A show ventral views of the superficial plane of the stratigraphic dissection (only skin was removed). Figures 1B, 4B and 7B show ventral views of the stratigraphically dissected heads: 1) after removal of musculus constrictor colli and musculus intermandibularis, and, 2) at a deeper dissection plane (the deeper dissection plane is marked on the figure). Two dissection planes – superficial, where only skin was removed (Figures 2A, 5A, 8A), and deep (Figures 2B, 5B, 8B) – are shown in LL views of the stratigraphically dissected heads.

The bony structures were clearly defined on the radiographs of all species (Figures 1C, 2C, 4C, 5C, 7C, 8C). Some soft tissues, such as the oesophagus, the trachea and the masticatory muscles, could be also identified.

A selection of matched cross-sections and CT images are shown in Figures 3, 6 and 9. In Figures 3A, 6A and 9A, the lines superimposed on the photographs indicate the approximate level of the matched images displayed in the corresponding figures. The level of the cross-sections displayed in the images is similar for all the species considered in order to emphasise the comparative imaging features. A small amount of fluid was noticed in the oesophagus of the cross-sectioned iguanas (Figure 3H).

## Discussion

The skull of lizards belonging to the infraorder *Iguania* is roughly triangular in dorsal view, with a short pre-orbital region. It retains all the characteristics of the ancestral lizard skull with no secondary closure of the skull openings [15]. The main difference between *Iguanidae* and *Agamidae* is in the nature of tooth implantation – acrodontal rather than pleurodontal – although most agamids have at least some pleurodont teeth in the front lower jaw [15].

Standard positioning is a prerequisite for good film quality [29]. As in snakes, the junctions between the bones composing the lower jaw are relatively loose [13]. Therefore, radiographic positioning quality should be evaluated, in our opinion, primarily through the symmetry and superimposing of the bilateral structures of the snout and neurocranium both in lateral and sagittal projections.

All the radiographs and CT images shown in the figures were obtained from live animals. It is the authors’ opinion that, although a direct comparison between anatomical and diagnostic images was not possible, a significant correlation in the matched images was achieved. Moreover, the use of contrast medium enabled good visualisation of the soft tissues and overcame the lack of soft tissue detail encountered in other similar works performed only on cadavers [8].

The CT images of the iguana (Figure 3) provided good detail when displayed both in bone and soft tissue windows. The CT images of the tegu (Figure 6) displayed in a bone window provided good detail of the bony structures whereas in the images displayed in a soft tissue window only the eyes were clearly distinguishable from the remaining soft tissues. The CT images of the bearded dragon (Figure 9) were of a relatively lower quality in both the bone and soft tissue windows. This lack of detail is due to the small size of this species and to the impossibility to reduce the field of view of our CT scanner to less than 16 cm. Despite this lack of detail, most of the bony structures were identified in CT images displayed in the bone window. In the CT images displayed in the soft tissue window, only the eyes and the Harderian glands were distinguishable from the other soft tissues of the head.

The nasal cavity of all three species was clearly visible both in VD (Figures 1C, 4C, 7C) and LL (Figures 2C, 5C, 8C) radiographs. The septomaxilla and the nasal glands were very prominent in the LL radiographic projection of the iguana (Figure 2C), giving the nasal cavities an overall higher radiopacity than in the tegu (Figure 5C) and the bearded dragon (Figure 8C). The nasal cavity of the iguana appeared almost entirely occupied by the nasal glands both in cross-sections and CT scans (Figures 6B, C, D). The nasal glands in the bearded dragon (Figures 9B, C, D) were similar in appearance but less prominent in the nasal cavity. The nasal glands in the tegu were more medially located and less evident both in cross-sections and in CT images (Figures 6B, C, D).

The scleral ossicles were clearly visible both in the iguana (Figure 2C) and the tegu (Figure 5C) in LL radiographic projections, whereas they appeared less evident in the LL radiographic projection involving the bearded dragon (Figure 8C). Furthermore, the scleral ossicles were evident in CT images of all the examined species (Figures 3F,G, 6F,G, 9F,G) but were hardly visible in anatomic cross-sections (Figures 3E, 6E, 9E).

The bones composing the lower jaw were not individually evident either in LL or VD radiographic projections in any of the examined species because the sutures between the bones of the lower jaw are smaller than the minimum radiologic resolution.

The oesophagus was well defined in LL radiographs of all the examined species (Figures 2C, 5C, 8C). It appeared as a U-shaped radiolucency bordering the caudal aspect of the lower jaw in the iguana (Figure 2C) and bearded dragon (Figure 8C) whereas it appeared straighter in the tegu (Figure 5C). Furthermore, the dorsal portion of the oesophagus is partially superimposed on the highly developed masticatory muscles in the iguana and bearded dragon (Figures 2A and 8A, respectively) and is grossly much larger, as can be seen in the radiographs.

The trachea was clearly identified in the LL radiographic projection partially superimposed on the ventral aspect of the oesophagus in the iguana (Figure 2C) and tegu (Figure 5C), while it was difficult to identify in the bearded dragon (Figure 8C). In the iguana (Figure 2C) the trachea showed a peculiar outline turning two ninety-degree angles at the inlet of the coelomic cavity. All the animals were deeply sedated during the imaging procedures; the authors are unable to determine whether this latter feature is due to muscular relaxation induced by anaesthesia or is normal even in unsedated iguanas.

The eyes were very evident in all the species examined, both in cross-sections and CT images displayed in a soft tissue window (Figures 3E, G, 6E, G, 9E, G). The lens and the vitreous were clearly delineated both in cross-sections and CT images in the iguana (Figures 3E, G) and tegu (Figures 6E, G). The lens and the vitreous were not individually identified either in cross-sections or CT images in the bearded dragon (Figures 9E, G). In the iguana the Harderian glands were hardly visible in cross-sections (Figure 3E) while they were very prominent and clearly distinguishable from the underlying *sinus orbitalis* in CT images (Figure 3G). A radiodense line was noticed on the ventral aspect of the *sinus orbitalis* in post-contrast CT images of the iguana displayed in a soft tissue window (Figure 3G). In the tegu the Harderian glands were clearly evident in cross-sections (Figure 6H) but were difficult to identify in the CT images (Figure 6L). In the bearded dragon the Harderian glands could be identified both in cross-sections (Figure 9E) and CT images (Figure 9G) but were less evident than in the iguana.

The head of lizards can be affected by several pathologies; abscesses, metabolic bone diseases, fractures, osteomyelitis, and neoplasia [30-34].

Bites from preys, traumas, foreign bodies, pyogenic infections may lead to the formation of abscesses in the head of lizards; this is especially true in captive specimens, as they are often immunocompromised and more prone to develop inflammation [31]. Successful treatment of the reptilian abscess is due to the complete removal of abscess cavity and surrounding fibrous capsule [31]. Therefore both radiographic and CT studies could be helpful to determine the extension and the number of involved structures for a good pre-surgical evaluation. Moreover, CT studies may become mandatory to achieve a correct diagnosis in deep abscesses that have no external protrusion.

Metabolic bone disease may result from different underlying pathologies in reptiles [32]. To this effect, conventional radiology and dual-energy x-ray absorptiometry (DXA) techniques of the head of lizards are obvious diagnostic tools, and may also successfully contribute to the follow up evaluation of the relative treatments [2,14,32].

Skull fractures of traumatic or pathological origin (i.e.: metabolic bone disease, neoplasia) are not uncommon in reptiles [34]. Plain or conventional radiology is not considered as the technique of choice to assess a skull fracture due to: 1) the relatively small size of the bones composing the skull, and, 2) the high risk of border effacement due to different tissues superimposition that is intrinsic to the technique. On the contrary it is the authors’ opinion that CT may be useful to correctly assess skull fractures in lizards and, more in general, in reptiles.

The mandibula and the maxilla are the most common sites of osteomyelitis in the head; changes in their bony structure include osteolysis and less frequently new bone formation [30]. Although plain radiographs may be sufficient to diagnose this pathology [30]; nevertheless, in our opinion, CT scans may provide additional information on the extension of the infectious process and aid in planning a correct therapy.

Head/skull neoplasia is considered as a quite common disease affecting reptiles [33]. Since lizard patients often require sedation for diagnostic imaging procedures [30], it is the authors’ opinion that, CT may be the imaging technique of choice if neoplasia of the head is suspected. The mayor advantages of CT are: the possibility to fully visualize the extension of the neoplastic process; and the possibility to perform CT guided fine needle aspirates or biopsies [35].

The skull of reptiles develops from a post-hatching cranium, the chondrocranium, to its definitive form in adults, the osteocranium [15]. Often the bones composing the chondrocranium have not reached full adult form [15]. All the tables presented in this work refer to adult animals and care should be taken when using these tables to interpret radiographs or CT studies of immature animals.

## Conclusions

The radiographic and CT features described in this work will form a useful basis for interpreting imaging studies of the head of the green Iguana, tegu and bearded dragon, as well as providing a more general anatomic reference for structures of the head of these species. Nevertheless, if this work is used in the interpretation of medical images from species different from those described in this work, the interspecific differences should be known and considered.

## Endnote

^a^This study was carried out under the Approval of the University of Padua Ethical Committee (CEASA): Protocol No. 41060; July 12, 2010.

## Abbreviations

LL, Left lateral; VD, Ventrodorsal; CT, Computed tomography.

## Competing interests

The authors declare that they have no competing interests.

## Authors’ contributions

TB: conceived the study, performed the radiographs and the CT scans, drafted the manuscript; PS: participated in the design of the study and drafted the manuscript; IAV, AM, EZ: performed the anatomical dissections, matched the anatomical images with the radiographs and the CT scans, performed the anatomical recognition of the structures indicated in the figures; AZ: conceived the study, supervised the selection of patients, drafted the manuscript. All authors read and approved the final manuscript.
